# The International Virus Bioinformatics Meeting 2020

**DOI:** 10.3390/v12121398

**Published:** 2020-12-06

**Authors:** Franziska Hufsky, Niko Beerenwinkel, Irmtraud M. Meyer, Simon Roux, Georgia May Cook, Cormac M. Kinsella, Kevin Lamkiewicz, Mike Marquet, David F. Nieuwenhuijse, Ingrida Olendraite, Sofia Paraskevopoulou, Francesca Young, Ronald Dijkman, Bashar Ibrahim, Jenna Kelly, Philippe Le Mercier, Manja Marz, Alban Ramette, Volker Thiel

**Affiliations:** 1 European Virus Bioinformatics Center, 07743 Jena, Germany; niko.beerenwinkel@bsse.ethz.ch (N.B.); irmtraud.meyer@mdc-berlin.de (I.M.M.); georgia.m.cook@gmail.com (G.M.C.); c.m.kinsella@amsterdamumc.nl (C.M.K.); Kevin.Lamkiewicz@uni-jena.de (K.L.); Mike.Marquet@med.uni-jena.de (M.M.); d.nieuwenhuijse@erasmusmc.nl (D.F.N.); io239@cam.ac.uk (I.O.); sofia.paraskevopoulou@charite.de (S.P.); ronald.dijkman@vetsuisse.unibe.ch (R.D.); bashar.ibrahim@uni-jena.de (B.I.); jenna.kelly@vetsuisse.unibe.ch (J.K.); philippe.lemercier@isb-sib.ch (P.L.M.); manja@uni-jena.de (M.M.); alban.ramette@ifik.unibe.ch (A.R.); volker.thiel@vetsuisse.unibe.ch (V.T.); 2 RNA Bioinformatics and High-Throughput Analysis, Friedrich Schiller University Jena, 07743 Jena, Germany; 3 Department of Biosystems Science and Engineering, ETH Zurich, 4058 Basel, Switzerland; 4 SIB Swiss Institute of Bioinformatics, 4058 Basel, Switzerland; 5 Max Delbrück Center for Molecular Medicine in the Helmholtz Association, Berlin Institute for Medical Systems Biology, 10115 Berlin, Germany; 6 Department of Biology, Chemistry and Pharmacy, Institute of Chemistry and Biochemistry, Freie Universität Berlin, 14195 Berlin, Germany; 7 Lawrence Berkeley National Laboratory, DOE Joint Genome Institute, Berkeley, CA 94720, USA; sroux@lbl.gov; 8 Department of Pathology, Division of Virology, University of Cambridge, Cambridge CB2 1TN, UK; 9 Laboratory of Experimental Virology, Department of Medical Microbiology and Infection Prevention, Amsterdam UMC, University of Amsterdam, 1105 AZ Amsterdam, The Netherlands; 10 CaSe Group, Institut für Infektionsmedizin und Krankenhaushygiene, Universitätsklinikum Jena, 07743 Jena, Germany; 11 Viroscience Department, Erasmus MC, 3015 GD Rotterdam, The Netherlands; 12 Institute of Virology, Charité-Universitätsmedizin Berlin, Corporate Member of Freie Universität Berlin, Humboldt-Universität zu Berlin, and Berlin Institute of Health, 10117 Berlin, Germany; 13 MRC-University of Glasgow Centre for Virus Research, Glasgow G61 1QH, UK; f.young.1@research.gla.ac.uk; 14 Institute of Virology and Immunology, University of Bern, 3012 Bern, Switzerland; 15 Department of Infectious Diseases and Pathobiology, Vetsuisse Faculty, University of Bern, 3012 Bern, Switzerland; 16 Institute for Infectious Diseases, University of Bern, 3012 Bern, Switzerland; 17 Centre for Applied Mathematics and Bioinformatics, Hawally 32093, Kuwait; 18 Department of Mathematics and Natural Sciences Gulf University for Science and Technology, Hawally 32093, Kuwait; 19 Swiss-Prot Group, SIB Swiss Institute of Bioinformatics, 1205 Geneva, Switzerland

**Keywords:** virology, virus bioinformatics, COVID-19, software, metagenomics, virome, viral diversity, identification, genome evolution, viral taxonomy

## Abstract

The International Virus Bioinformatics Meeting 2020 was originally planned to take place in Bern, Switzerland, in March 2020. However, the COVID-19 pandemic put a spoke in the wheel of almost all conferences to be held in 2020. After moving the conference to 8–9 October 2020, we got hit by the second wave and finally decided at short notice to go fully online. On the other hand, the pandemic has made us even more aware of the importance of accelerating research in viral bioinformatics. Advances in bioinformatics have led to improved approaches to investigate viral infections and outbreaks. The International Virus Bioinformatics Meeting 2020 has attracted approximately 120 experts in virology and bioinformatics from all over the world to join the two-day virtual meeting. Despite concerns being raised that virtual meetings lack possibilities for face-to-face discussion, the participants from this small community created a highly interactive scientific environment, engaging in lively and inspiring discussions and suggesting new research directions and questions. The meeting featured five invited and twelve contributed talks, on the four main topics: (1) proteome and RNAome of RNA viruses, (2) viral metagenomics and ecology, (3) virus evolution and classification and (4) viral infections and immunology. Further, the meeting featured 20 oral poster presentations, all of which focused on specific areas of virus bioinformatics. This report summarizes the main research findings and highlights presented at the meeting.

## 1. Introduction

The International Virus Bioinformatics Meeting (IVBM) is a newly conceived conference that has emerged from the Annual Meeting of the European Virus Bioinformatics Center (EVBC). The EVBC was founded in 2017 to bring together experts in virology and virus bioinformatics in Europe [1,2]. The EVBC is constantly growing, having currently 188 members from over 100 research institutes distributed over 30 countries worldwide.

In 2020, the IVBM was held for the first time, extending the Annual Meeting of the EVBC (see Table 1). The meeting was originally planned to take place in Bern, Switzerland, in March 2020. However, due the COVID-19 pandemic, the conference was rescheduled to 8–9 October 2020 and even switched to an online format at short notice.

The pandemic has made us even more aware of the importance of accelerating research in viral bioinformatics. IVBM 2020 attracted approximately 120 experts in virology and computational biology at all career stages to attend the two-day online meeting. Despite concerns being raised about the lack of opportunities for face-to-face discussions in virtual meetings, the participants created a highly interactive scientific environment, engaging in lively and inspiring discussions and suggesting new research directions and questions. A coffee break atmosphere was created in breakout rooms, with the speakers being available for continued discussion. The meeting featured five invited and twelve contributed talks, as well as 20 posters which were presented during a virtual poster session. Online recordings of the talks were watched afterwards by more than 100 people.

## 2. Sessions and Oral Presentations

A number of high-quality presentations were given by leading experts and junior scientists on the four main topics: proteome and RNAome of RNA viruses (see Section 2.1), viral metagenomics and ecology (see Section 2.2), virus evolution and classification (see Section 2.3) and viral infections and immunology (see Section 2.4). The meeting featured five invited talks and twelve talks that were selected from over 40 submissions.

The conference was opened with a talk by Volker Thiel (University of Bern, Bern, Switzerland) on “20 years of coronavirus reverse genetics”. Volker Thiel, who was one of the main organizers of the meeting, is a leading expert on coronaviruses, making his presentation the perfect opening in view of the ongoing pandemic and the resulting difficulties in organizing the conference.

### 2.1. Proteome and RNAome of RNA Viruses

This session was chaired by Philippe Le Mercier (University of Geneva Medical School, Geneva, Switzerland), one of the local organizers of the meeting and board member of the EVBC. Two speakers have been invited on this topic. Alexander Gorbalenya (Leiden University Medical Center, Leiden, The Netherlands), presented recent advancements in comparative genomics of RNA viruses. Irmtraud Meyer (Max Delbrück Center for Molecular Medicine in the Helmholtz Association, Berlin, Germany) presented computational methods for identifying functional RNA structural features. From the submitted abstracts, we selected talks by Ingrida Olendraite on expanding the diversity and molecular biology of RNA viruses; and Georgia Cook on ribosome profiling (RiboSeq) for analysing virus gene expression.

#### 2.1.1. Computational Methods for Identifying Functional RNA Structure Features—By Irmtraud M. Meyer

Most viral genomes share one common functional constraint and that is to keep their genome rather short for the sake of efficient packaging and swift replication. On the other hand, different viruses have to fulfil a range of diverse functional roles that reflect the variety of in-vivo constraints they face throughout their different stages of infection in different hosts. All viral genomes thus also have a pressing need to encode a variety of functional information that they require at different life stages into their rather short genomes. The emerging view, however, is that the functional range of any virus is not only determined by its encoded protein-coding genes, but also by other types of functional features that are harder to discern, e.g., functional RNA structural features.

Due to the degeneracy of the genetic code, any protein-coding region can also encode overlapping RNA structural information. This even applied to overlapping open-reading frames such as those encountered in HIV [5]. As they have shown in the past [6,7], however, it is vital to correctly capture the known protein-context when trying to computationally detect any (partially or completely) overlapping RNA structural feature. This can only be achieved via dedicated (and -typically -complex) models of RNA structural features that are able to integrate prior information on protein-coding regions into a joint, mathematically principled predictive framework. This has recently allowed them the computational identification of local RNA structural features that are key for regulating functionally important alternative splicing in influenza A [8].

On the computational side, RNA structure prediction—especially in the context of pathogens—is further complicated by the fact that (1) different life stages of the pathogen require different functional signals and that (2) the expression of any particular RNA structural feature in vivo typically strongly depends on its complex molecular context, e.g., RNA-binding proteins, other trans interaction partners and also the kinetics of RNA sequence synthesis [9,10,11]. To tackle these conceptual challenges, the Meyer group has developed computational methods that allow functionally relevant RNA structures to be identified and visualised beyond the one-sequence-one-structure dogma, e.g., [12,13,14,15]. These methods focus on identifying the RNA structure(s) that have been conserved throughput well-chosen times of evolution rather than predicting the thermodynamically most stable RNA structure, which may only be relevant in a more-artificial in-vitro setting.

The last few years have seen an exciting range of new experimental high-throughput methods for probing the RNA structurome and RNA–RNA interactome by detecting so-called duplexes in vivo [16,17,18]. The raw data generated by these methods currently require fairly sophisticated computational analyses for pre-processing and interpretation in terms of distinct RNA structures and trans RNA–RNA interactions and also come with a range of experimental biases that need to be accounted for [19]. In the near future, especially when combined with established SHAPE-probing methods, these novel methods should give us significant new biological insight into functionally relevant RNA structures and trans RNA–RNA interactions in the context of pathogen–host interactions. The Meyer group has recently updated its well-known R-Chie visualisation web-server (https://e-rna.org/r-chie) to now cater not only for RNA structures, but also for *trans* RNA–RNA interactions and genome interactions [15,20,21].

#### 2.1.2. Expanding Diversity and Molecular Biology of RNA Viruses—By Ingrida Olendraite

*Ingrida Olendraite received the Best Newcomer Talk Award.*

RNA viruses are very diverse, have high mutational rates, and employ an enormous variety of molecular strategies to transcribe and translate their genes. While human-infecting viruses have been well-characterized, such viruses make up only a small proportion of natural RNA virus diversity. To better understand the evolution of RNA viruses and the molecular mechanisms that they employ, we were bioinformatically exploring the RNA viromes of diverse host organisms (over 1000 different species).

The viral RNA-dependent RNA polymerase (RdRp) is the only protein which is common to all RNA viruses. Thus, we can at least partly uncover global RNA virus diversity by finding their RdRp sequences [22]. When very divergent viruses are identified, we can propose new virus families and potentially make predictions about their gene expression mechanisms [23].

**Figure 1:**
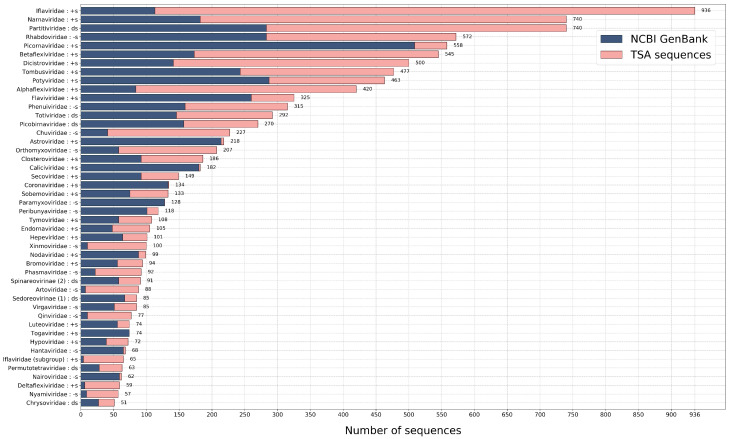
The number of identified non-identical RdRp sequences. Each identified sequence with high score was trimmed to the RdRp core and then sorted into the genetically most similar group (the best HMM profile match). The 100% identical sequences within a group have been removed. Here, we only show groups, where the total number of sequences left are above 50. The genome type of each group is identified as follows: +s is positive single-stranded, -s is negative single-stranded and ds is double-stranded RNA genome. The source of the sequences is identified by different colours.

Therefore, we created and used 77 Hidden Markov Model profiles, to search and identify viral RdRps among viral NCBI sequences and in 2649 transcriptomic datasets (see Figure 1). We have identified over 10,000 RdRps (half of which are new). Using these RdRps, we showed family-level evolutionary relationships and enriched diversity within numerous virus (mostly family level) groups. Moreover, some highly divergent and novel viral sequences have been identified and the potential host diversity has been expanded to new and some existing RNA virus groups.

#### 2.1.3. Using Ribosome Profiling (RiboSeq) as a Tool to Analyse Virus Gene Expression—By Georgia Cook

*Georgia M. Cook, Katherine Brown, Pengcheng Shang, Yanhua Li, Sawsan Napthine, Adam Dinan, Ying Fang, Andrew E. Firth, Ian Brierley have contributed to this work.*

Ribosome profiling (RiboSeq) is a next-generation sequencing-based technique which allows the positions of translating ribosomes to be mapped to a genome with sub-codon precision [24,25].

**Figure 2:**
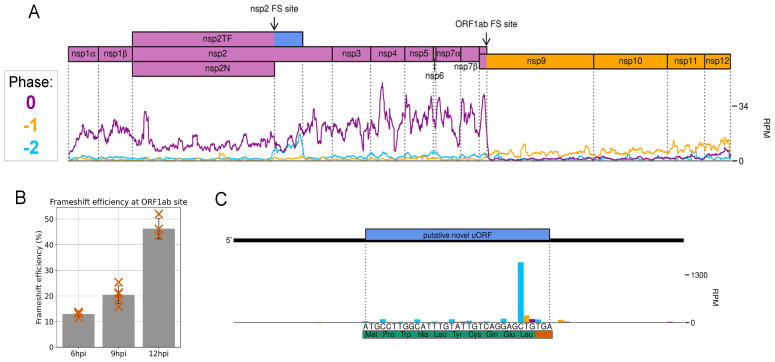
Ribosome profiling of PRRSV-2, displaying reads mapped to the polyprotein region of the genome. (**A**) Read density at each position, after application of a 27-codon sliding window running mean, coloured according to phase (codon position to which the 5${}^{\prime }$ end of a read maps). An increase in the proportion of reads in the −2 phase (blue) is visible in the transframe region directly following the nsp2 frameshift site. The dominant phase returns to 0 (purple) after the stop codon in the −2 frame causes termination of frameshifted ribosomes. After the ORF1ab frameshift site, the ribosome density decreases, as ribosomes which do not frameshift encounter a stop codon, and ribosomes which undergo −1 PRF continue translation in the −1 frame, resulting in reads predominantly in the −1 phase (yellow). (**B**) Frameshift efficiency at the ORF1ab site increases as infection progresses. (**C**) A small but highly expressed upstream ORF (uORF) in the 5${}^{\prime }$UTR of the PRRSV genome. The prominent peak corresponding to terminating ribosomes is characteristic of samples harvested without drug pre-treatment.

We carried out this analysis on samples derived from an infection of MARC-145 cells with the economically important arterivirus, porcine reproductive and respiratory syndrome virus (PRRSV), harvested over a timecourse of infection. The PRRSV genome is unusual in that it contains two programmed ribosomal frameshift (PRF) sites, which promote slippage of a proportion of translating ribosomes by 1 or 2 nt backwards, after which decoding continues in alternative reading frames [26,27,28]. The distance between the 5${}^{\prime }$ end of RiboSeq reads and the first nucleotide of the ribosomal P site was determined, allowing reads to be mapped to the genome with sub-codon resolution and thus the frame of translation inferred. This permitted visualisation of changes in reading frame downstream of both PRF sites on the PRRSV genome (see Figure 2A), which became more conspicuous when read counts on the WT viral genome were normalised by those of a frameshift-defective mutant.

Analysis of the cardiovirus Theiler’s murine encephalomyelitis virus (TMEV), which also utilises PRF to regulate viral gene expression [29], demonstrated a decreased read density in the region downstream of the PRF site, as a result of ribosomes terminating translation upon encountering an early stop codon in the alternative reading frame. Comparison of read density upstream and downstream of the frameshift site revealed that the frameshift efficiency is ~85%, the most efficient known natural example of −1 PRF [30].

Calculations for PRRSV revealed that −1 PRF efficiency increases as infection progresses (see Figure 2B), apparently overturning the common assumption that PRF stimulated by RNA secondary structures occurs at a fixed efficiency.

Ribosome profiling can also reveal novel features of the viral translatome. For example, we discovered a short but conserved and highly expressed upstream ORF in the 5${}^{\prime }$UTR of the PRRSV genome (see Figure 2C).

### 2.2. Viral Metagenomics and Ecology

This session was chaired by Alban Ramette (University of Bern, Switzerland), one of the local organizers of the meeting. One speaker has been invited on this topic: Simon Roux (DOE Joint Genome Institute, Berkeley, CA, USA) presented how to explore viral diversity and virus–host interactions from metagenomes. From the submitted abstracts, we selected talks by Pauline Dianne Santos (Friedrich Loeffler Institute, Greifswald, Germany) on metagenomics analyses of West Nile virus outbreak samples from Germany; David Nieuwenhuijse (Erasmus University Medical Center, Rotterdam, The Netherlands) on browsing virome sequencing analysis results; Olivier Zablocki (Ohio State University, Columbus, OH, USA) on enabling low-input, long-read viromics using VirION2; and John Beaulaurier (Oxford Nanopore Technologies, San Francisco, CA, USA) on unsupervised clustering of nanopore reads producing thousands of complete phage genomes from marine samples.

#### 2.2.1. Viral Ecogenomics: Exploring Viral Diversity and Virus–Host Interactions from Metagenomes—By Simon Roux

Microbes are recognized as playing key roles in all ecosystems, driving nutrient and energy transfers, and directly influencing human health and disease. While microbes are the principal and most-studied components of microbiomes, all microbial processes are strongly constrained and altered by viruses [31,32,33]. In terms of numbers alone, virus-like particles seemingly outnumber microbial cells in every ecosystem. The world’s oceans, for example, harbor an estimated $10^{30}$ virus particles, with an estimated one out of three cells infected at any given time. The most intuitive impact of these many viral infections is virus-induced mortality of microbial cells, which can trigger large-scale reshuffling of microbial communities [34,35]. However, viruses can also modify their host cell metabolism and alter host cell fitness, including during latent and/or chronic infections [31,36,37]. Understanding these different virus–host interactions and their associated ecological and evolutionary drivers is thus critical to fully comprehend microbiome dynamics.

**Figure 3:**
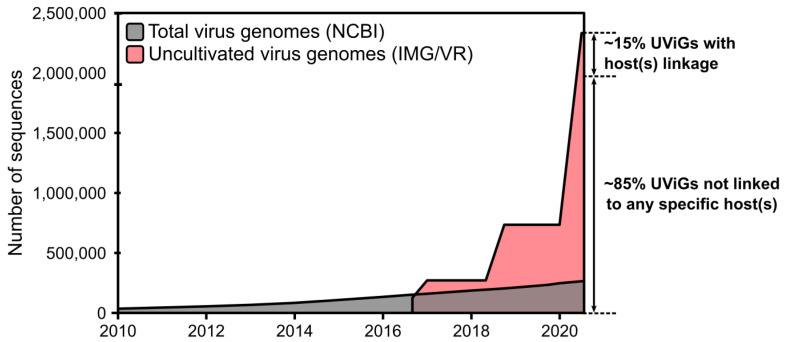
Growth of virus genome databases from 2003 to 2020. The total number of genomes from isolates was based on queries to the NCBI nucleotide database portal, while the number of uncultivated virus genomes (UViGs) was estimated by compiling data from the literature and from the IMG/VR database, as in Paez-Espino et al. [38].

Thus far, technical challenges have limited our ability to even catalog the global virosphere, leading to the denomination of these seen-but-uncharacterized viruses as “dark matter of the biological universe”. In the past three years alone however, metagenomic approaches increased viral genome databases by >200 times, and enabled comparative genomics studies, which have already revealed ≥900 new candidate viral genera (see Figure 3) [38,39,40]. While still incomplete and not yet evenly representing the true extent of viral diversity in nature, this comprehensive catalog of uncultivated viral genomes represents an invaluable resource to evaluate ecological and evolutionary patterns in the viral world. In addition, exploring the functional potential of uncultivated viruses can suggest new putative mechanisms by which viruses can manipulate microbial processes [41,42].

Our current work involves the development of new approaches to maximize the recovery of viral genomes from metagenomes and to make these bioinformatic tools available to the broader community of researchers. As part of the growing viral ecogenomics community, we recently outlined the current promises and pitfalls of these analyses and established the first standards to report viral genomes assembled from metagenomes (“Minimum Information about an Uncultivated Virus Genome (MIUViG)”) [43]. We also recently demonstrated how customized machine-learning-based techniques could reveal an extensive viral diversity “hidden” in publicly available genomic and metagenomic datasets, by vastly expanding the genome diversity and host range of a family of bacteriophages (Inoviridae) [44]. Finally, we are currently exploring the use of targeted metagenomics and time-series analysis to understand the eco-evolutionary drivers and constraints on virus–host dynamics in nature. Eventually, we envision that a full viral ecogenomics toolkit will soon empower researchers to scrutinize viral communities and virus–host interactions with an unprecedented level of detail and resolution, enabling us to revisit long-standing biological questions and possibly inspiring new technologies for microbiome manipulation [45,46,47,48].

#### 2.2.2. viromeBrowser: A Shiny App for Browsing Virome Sequencing Analysis Results—By David Nieuwenhuijse

Experiments in which complex viromes are sequenced generate data that are difficult to visualize and unpack for virology experts who do not have bioinformatics expertise. After processing raw sequencing data, generated by next generation sequencing (NGS) workflows, the output often consists of contiguous sequences (contigs) in FASTA format. Usually, these contigs are combined with an annotation file linking the contigs to a reference sequence or taxonomic identifier in tabular format, accompanied by several annotation quality parameters. For most bioinformatic NGS workflows, this is the end result; however, in these types of experiments, the next step is to visually inspect the annotation result and to filter any miss-annotations by hand. In addition, the results of different samples can be compared based on their metadata and sequences of interest can be extracted for use in subsequent analyses. In the case of complex virome NGS data, this is not a trivial job because these data contain many contigs with various levels of annotation quality potentially spread over multiple files, making it difficult to obtain a good overview of the data. Moreover, depending on the research question of the user (detection, complete genome sequencing or virus discovery), annotation quality thresholds need to be modified. To aid virology experts in the inspection of complex virome NGS data, we have developed viromeBrowser, a web tool which is focused on integrating viral sequence data, annotation data and sample metadata and facilitates interactive data browsing with a user-friendly interface.

The viromeBrowser facilitates the browsing of complex virome data by dividing this process into multiple steps. First the annotation data, sequence data and metadata are loaded into the app, after which the different quality parameters of the annotation data can be set to the user’s preference or can be left on a default mode. Second, hypothesis-based selections of the data can be made according to the provided metadata. After filtering, contigs of interest can be selected and downloaded or further inspected by prediction of the open reading frames (ORF) and visualization of nucleotide abundance. The viromeBrowser was implemented using the R Shiny platform (RStudio I. shiny: Web Application Framework for R [Internet]. 2020. Available from https://shiny.rstudio.com/) which is used to create interactive web applications. The server-client architecture of shiny apps allows for heavy lifting on the server side and visualization of the results on the client side. Moreover, shiny modules can be easily copied and used elsewhere, making it possible for other developers to build upon it. Other metagenome data visualization tools are available, such as MEGAN [49], Krona [50], Anvi’o [51] and CLC bio, but these tools either focus on bacterial metagenomes, cannot be easily adapted, or require a payed license to use.

The viromeBrowser enables virology experts with little programming experience to interactively browse their virome NGS analysis results. In the viromeBrowser app, users can flexibly set annotation quality thresholds depending on their preferences and filter and compare samples based on metadata associated with the sample. The viromeBrowser is written in the opensource R shiny framework, making it free and easy for other shiny developers to expand.

### 2.3. Virus Evolution and Classification

This session was chaired by Manja Marz (Friedrich Schiller University Jena, Jena, Germany), one of the organizers of the meeting and board member of the EVBC. One speaker was invited on this topic: Niko Beerenwinkel (ETH Zurich, Zurich, Switzerland) presented on leveraging high-throughput sequencing data to investigate viral diversity. From the submitted abstracts, we selected talks by Mike Marquet (Jena University Hospital, Jena, Germany) on a parallel and scalable workflow for the identification and analysis of phages in sequencing data; Sofia Paraskevopoulou (Charité—Universitätsmedizin Berlin, Berlin, Germany) on re-assessing the diversity of negative strand RNA viruses in insects; and Kevin Lamkiewicz (Friedrich Schiller University Jena, Jena, Germany) on comparative genomics based on viral clusters.

#### 2.3.1. Leveraging High-Throughput Sequencing Data to Investigate Viral Diversity—By Niko Beerenwinkel

**Figure 4:**
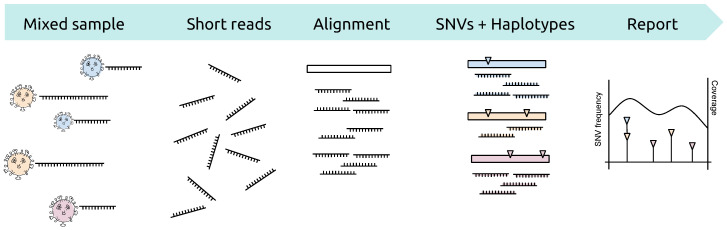
Basic workflow of V-pipe for end-to-end analysis of viral NGS data. Starting from a heterogeneous virus population, amplification and sequencing will produce a set of sequencing reads, each derived from one of the molecules in the original mixed sample. Reads are aligned, possibly with the help of a reference genome, and filtered according to various quality control criteria. From the multiple alignment of all reads, genetic variants are called either position-wise (single-nucleotide variants, SNVs) or for longer genomic regions (viral haplotype reconstruction). The results, including all SNVs and their estimated frequencies in the virus population, are summarized and visualized in an electronic interactive report.

RNA viruses can display a large amount of genetic diversity within a single infected host. This genetic heterogeneity is the result of high mutation rates, short generation times, and large population sizes. It is shaped by various selective pressures, including host immune responses and antiviral therapy. Intra-host genetic diversity has been associated with disease progression, drug resistance development, and immune escape. It can also be informative of infection dynamics, transmission chains, and selection pressures. However, assessing viral genetic diversity from next-generation sequencing (NGS) data remains challenging due to short read length and amplification and sequencing errors. We have developed V-pipe [52], a robust computational pipeline for automated end-to-end analysis of viral NGS data. V-pipe comprises steps for quality control, read alignment, single-nucleotide variant (SNV) calling, viral haplotype inference, and visualization by integrating different computational tools and by developing new tools (see Figure 4). The pipeline is designed in a modular fashion and can be adapted to different viruses, experimental designs, and sequencing technologies. It enables fully reproducible, transparent, and traceable viral NGS data analysis and quantification of viral genetic diversity. V-pipe includes modules for testing and benchmarking, thereby supporting the development and validation of novel viral NGS data analysis workflows. V-pipe is an actively maintained, open source, community-driven software project (https://cbg-ethz.github.io/V-pipe/). We applied V-pipe to 4000 deep-coverage SARS-CoV-2 samples to survey, in a comprehensive fashion, the intra-host genomic diversity of the virus causing the COVID-19 pandemic [53]. We assessed genomic heterogeneity on the level of individual SNVs, consecutive stretches of base pairs, and on the level of genes. We found that SARS-CoV-2 displays considerable genomic diversity, especially in the M gene, and that most of the genetic heterogeneity occurs in few samples and at few loci.

#### 2.3.2. Parallel and Scalable Workflow for the Identification and Analysis of Phages in Sequencing Data—By Mike Marquet

*Mike Marquet, Mathias W. Pletz, Oliwia Makarewicz, Martin Hölzer, Adrian Viehweger, Ralf Ehricht and Christian Brandt have contributed to this work.*

Phages will be increasingly used as platforms for antigen display, in pathogen detection, or as vaccines, e.g., as an alternative for the treatment of multiresistant bacteria. Long read sequencing technologies, such as nanopore sequencing, allows for complete phage-genomes sequencing anytime, with comparatively low investment. Sequencing data, in general, allows us to study the occurrence, spread and the type of bacteriophages, which in return, increases the demand for automated pipelines. A variety of bacteriophage identification tools have been developed over the years. They differ in algorithmic approach, results and ease of use. We, therefore, developed “What the Phage” (WtP), an easy-to-use and parallel multitool approach for phage identification combined with an annotation and classification downstream strategy, thus, supporting the user’s decision-making process when the phage identification tools are not in agreement to each other.

WtP is written in Nextflow and utilizes Docker and Singularity containers for a simplistic workflow execution in any Linux environment. All containers are written, tested and stored on Docker Hub (https://hub.docker.com/u/multifractal). WtP automatically utilizes these containers which in return means that the Installation of specific tools is no longer needed. All dependencies or databases are automatically downloaded. WtP is freely available via GitHub (https://github.com/replikation/What_the_Phage), which includes a simple installation routine for Nextflow/Docker.

We established a reproducible, scalable and easy-to-use workflow for phage identification and analysis. Our tool currently combines eleven established phage identification tools: Marvel, Virfinder, PPR-Meta, Virsorter, Metaphinder, DeepVirFinder, Sourmash, Vibrant, Virnet, Phigaro, Virsorter2(beta). WtP analyses and summarizes the results gathered from sequencing data. For this, each sample is combined into heatmaps for comfortable results interpretation. Moreover, multiple samples are computed in parallel and if less hardware is available, WtP decreases the parallelization automatically.

**Figure 5:**
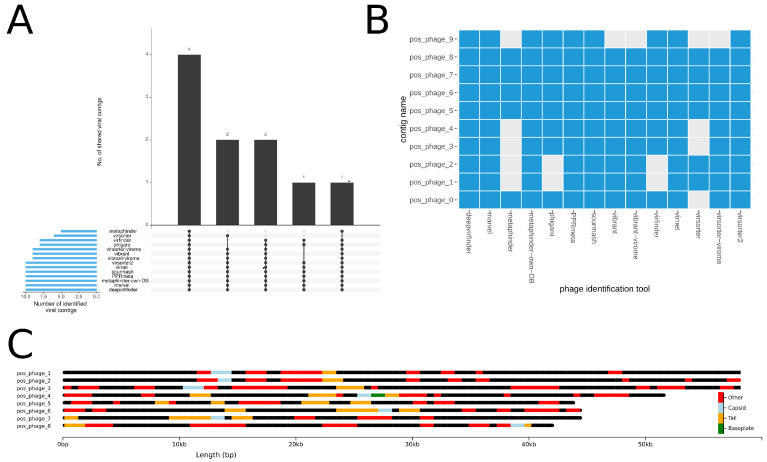
(**A**) The UpSet plot is summarizing the identification performance of each tool for a multi fasta sample. The total amount of identified phage-contigs per tool is shown in blue bars on the left. Black bars visualize the number of contigs that each tool or tool combination has uniquely identified. Each tool combination is shown below the barplot as a dot matrix. (**B**) Modified heatmap for phage sequences visualising the tool agreements per phage positive contig. (**C**) Visual annotation of phage contigs and annotated protein-coding genes via chromoMap. Annotations are coloured based on the categories of capsid genes (blue), tail genes (orange), baseplate genes (green) and other phage genes (red). For better readability, the contigs pos_phage_0 and pos_phage_9 were removed from the chromomap.

WtP is a highly robust and stable pipeline for the identification and analysis of phages which can easily handle both single and multi-sample inputs. After a WtP run, the user is provided with sufficient processed data (such as tool performance comparisons, taxonomic assessments, and annotation maps) to reliably work with the identified sequences (see Figure 5).

#### 2.3.3. Re-Assessing the Diversity of Negative Strand RNA Viruses in Insects—By Sofia Paraskevopoulou

*Simon Käfer, Sofia Paraskevopoulou, Florian Zirkel, Nicolas Wieseke, Alexander Donath, Malte Petersen, Terry C. Jones, Shanlin Liu, Xin Zhou, Martin Middendorf, Sandra Junglen, Bernhard Misof, and Christian Drosten have contributed to this work.*

The spectrum of viruses in insects [54] is important for subjects as diverse as public health, veterinary medicine, food production, and biodiversity conservation. The traditional interest in vector-borne diseases of humans and livestock has drawn the attention of virus studies to hematophagous insect species. However, these represent only a tiny fraction of the broad diversity of Hexapoda, the most speciose group of animals. Our work is focused on computational assessment of the diversity of negative strand RNA viruses within the largest and most representative collection of insect transcriptomes, from individuals representing all 34 extant orders of Hexapoda and several outgroups, altogether representing 1243 species. These transcriptomes were sequenced within the 1KITE project [55] from individuals that belong to all orders of Insecta (n=1178), to Collembola (n=23), Protura (n=4), and Diplura (n=14) of Entognatha, as well as 24 outgroup species that belong to Crustacea (n=10), Myriapoda (n=11), and Chelicerata (n=3).

Our search relied on the conserved sequence motifs within the RNA-dependent RNA polymerase (RdRp) gene that is present in the genomes of all replicating negative-strand RNA viruses without a DNA stage, except deltaviruses. We built profile hidden Markov models [56] to search for candidate viral RdRp motifs within 42,618,061 contigs and scaffolds. The models utilized template amino acid alignments covering the core conserved RdRp region of viruses assigned to four families and nine genera that belong to the orders of *Bunyavirales*, *Articulavirales*, and *Mononegavirales*.

**Figure 6:**
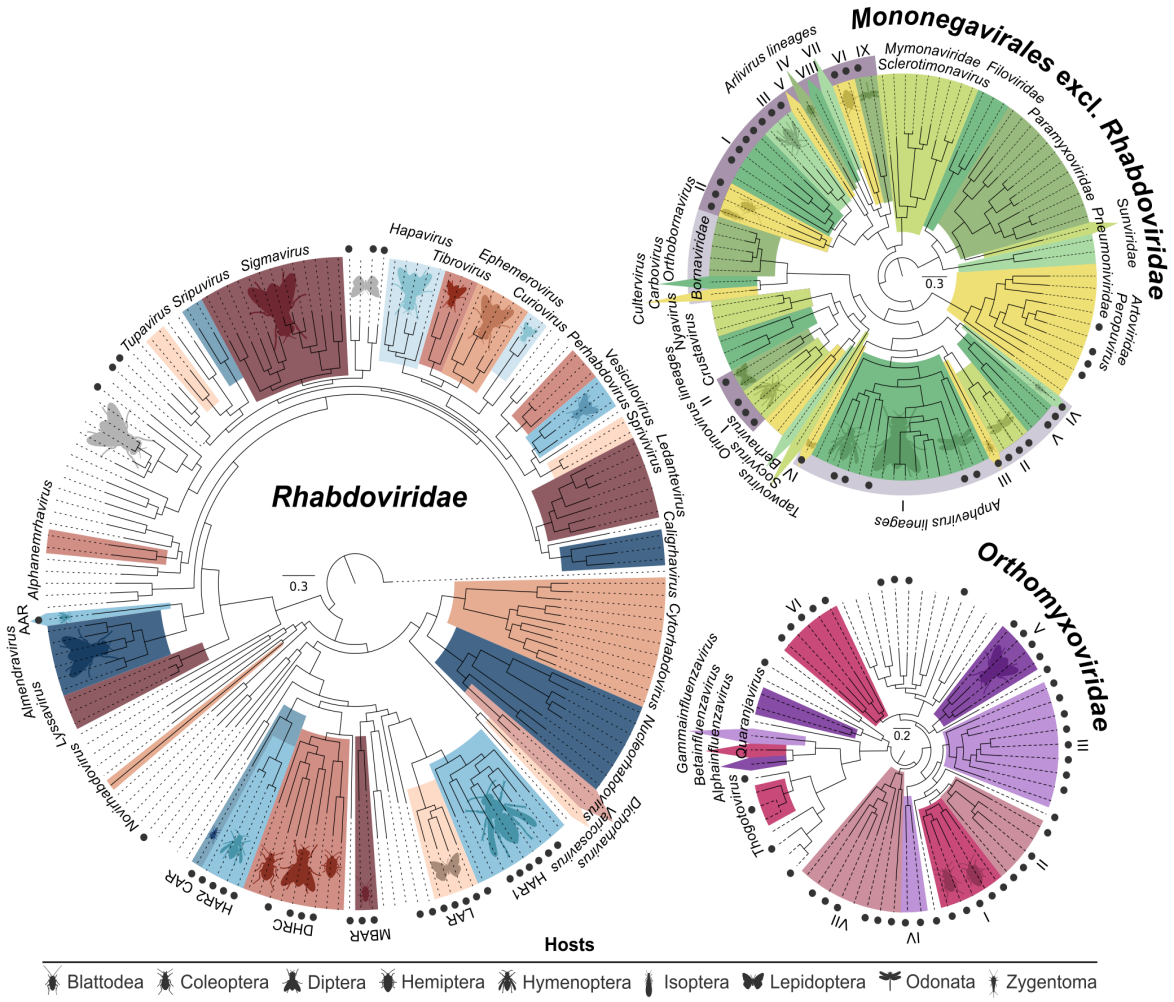
Maximum-likelihood phylogenies of viruses found in our study [54], classified viruses by ICTV, as well as selected unclassified viruses. Novel viruses from our study are identified by black circles. Abbreviations for the *Rhabdoviridae* phylogeny are: AAR—Almendra-related virus, CAR—Coleoptera-related viruses, HAR1 and −2—Hymenoptera-related viruses, DHRC—Diptera-, Hymenoptera-, and Coleoptera-related viruses, LAR—Lepidoptera-related viruses, MBAR—Mantodea-, and Blattodea-related viruses. The *Orthomyxoviridae* and the *Mononegavirales* excl. *Rhabdoviridae* phylogenies carry annotations in roman numbers for the different *Orthomyxoviridae* lineages, and *Arlivirus*, *Orinovirus*, and *Anphevirus* lineages that are defined after the addition of our data. Insect host orders relative to clades are watermarked wherever applicable.

Initially, 488 viral RdRp sequences in 324 arthropod species were detected, with similarity to negative strand RNA viruses. From the obtained contigs, 234 large sequences were selected for further analyses based on length, quality, and dissimilarity toward other sequences in the dataset. These sequences showed highest similarity to members of *Bunyavirales* (n=86), *Articulavirales* (n=54), or *Haploviricotina* (n=94), respectively. For viruses with segmented genomes, assembly focused on the RdRp-encoding segment was complemented by BLAST-based searches for other genome segments, resulting in 218 coding sequences from genes that are not encoded on the same segment as the RdRp gene. Coding-complete genomes or nearly-complete subgenomic assemblies were obtained in 61 cases. Figure 6 shows the phylogenetic trees of viruses pertaining to *Mononegavirales* and *Orthomyxoviridae*.

The discovery of a large diversity of novel lineages warrants a re-assessment of the overall topology of negative strand RNA viruses. Based on phylogenetic topologies and the cophylogenetic segregation signal for different genomic segments, the availability of coding-complete genomes, and host associations of the viral sequences, we estimate that at least 20 novel viral genera in seven families need to be defined, only two of them monospecific. Seven additional viral clades emerge when adding sequences from the present study to formerly monospecific lineages, potentially requiring the taxonomic assignment of up to seven additional genera altogether to these lineages. Considering the shortcomings of evolutionary distance as a single classification criterion, additional biological criteria should be included in taxonomic considerations (i.e., host associations), if only as a test of plausibility.

#### 2.3.4. Reducing Haystacks to Needles: Comparative Genomics Based on Viral Clusters—By Kevin Lamkiewicz

Most viruses are still unknown. However, for some species like *Influenza A virus* or HIV, we are faced with an enormous amount of data, having databases with up to millions (and sometimes redundant) sequence entries.

To provide confident insights into conserved elements of viral genomes, researchers usually base their analyses on multiple sequence alignments (MSA) of closely related genomes. However, building MSAs on hundreds of thousands of sequences is not practicable in terms of computational time, memory or storage, whereas picking representative sequences is not trivial.

Here, we present the Nextflow [57] pipeline ViralClust that can deal with a vast amount of viral sequences and assigns each virus to a cluster.Four approaches are currently implemented as modules, one of them being a novel approach of clustering viral genomes based on kmer frequencies. For this, we employ the methods UMAP [58] and HDBSCAN [59] to represent each genome as a vector in a high-dimensional space. Nextflow is used to ensure reproducible and transparent analyses and results. ViralClust is still under development, however, an alpha version is available (https://github.com/klamkiew/viralClust).

First, redundant sequences are removed, and the positive strand of each viral genome is stored. Then, these filtered genomes are used to calculate clusters with each implemented method. For each cluster approach, ViralClust reports a set of representative genomes. If set, the user can generate a rough evaluation of the different cluster algorithms, including the number of calculated clusters, the minimum, maximum and average cluster size, and the number of unclustered sequences. For this, a multiple sequence alignment with MAFFT [60] and a phylogenetic tree with FastTree [61] are calculated and used to derive basic statistics.

We refrain from recommending one algorithm as the best since the evaluation of clustering results is highly dependent on the underlying scientific question. For example, for *Filoviridae* (single-stranded, negative-oriented RNA viruses) cd-hit-est [62] forms a large cluster for the species *Zaire ebolavirus*. On the other hand, our HDBSCAN approach finds more subtle changes, thus generating more clusters for *Zaire ebolavirus*. These clusters represent different outbreaks of Ebolavirus, for example, the most prominent in the Democratic Republic of Congo in 2014/2015. Thus, different scientific questions need different resolutions of the resulting cluster. With ViralClust, we aim to provide an easy-to-use pipeline that assists the user with the correct choice of representative genomes for their specific question.

With our in silico pipeline, we can determine a small set of representative viruses based on millions of different strains and species. Thus, the process of selecting genomes for multiple sequence alignments and further downstream analyses is made easily accessible and may improve comparative genomics for viral genomes.

### 2.4. Viral Infections and Immunology

This session was chaired by Volker Thiel. One speaker has been invited on this topic: Daniel Blanco Melo (Icahn School of Medicine at Mount Sinai, New York, NY, USA) presented how the imbalanced host response to SARS-CoV-2 drives the development of COVID-19. From the submitted abstracts, we selected talks by Francesca Young (MRC-University of Glasgow Centre fo Virus Research, Glasgow, UK) on a machine learning approach to predict host taxonomic information from viral genomes; Cormac M. Kinsella (Amsterdam UMC, Amsterdam, The Netherlands) on recombination networks and endogenous viral anchors for high-throughput host identification; and Neta Zuckerman (Sheba Hospital, Ramat Gan, Israel) on single cell molecular dynamics in mice infected with West Nile virus.

#### 2.4.1. Machine Learning Approach to Predicting Host Taxonomic Information from Viral Genomes: Combining Feature Representations—By Francesca Young

Knowing the host of a virus is important, both in the case of identifying the source of a newly emerged pathogen, such as SARS-CoV-2, or for understanding the impact of virus–host relationships within a microbiome. Continued advances in metagenomics has resulted in an unprecedented growth in viral discovery but the majority of these new sequences have no assigned host. Current computational approaches to predicting virus host infection tend to be based on genome sequence similarity approaches or machine learning methods that use nucleotide signatures as features. There is scope for developing a wider range of features, encapsulating other levels of biological information. This study is based on the premise that a host-specific signature is embedded in viral genomes by the process of virus host coevolution, mimicry of host genome patterns and specific virus–host molecular interactions. Our goal was to investigate the predictive potential of features generated from different levels of viral genome representation to encapsulate these host-specific signals.

**Figure 7:**
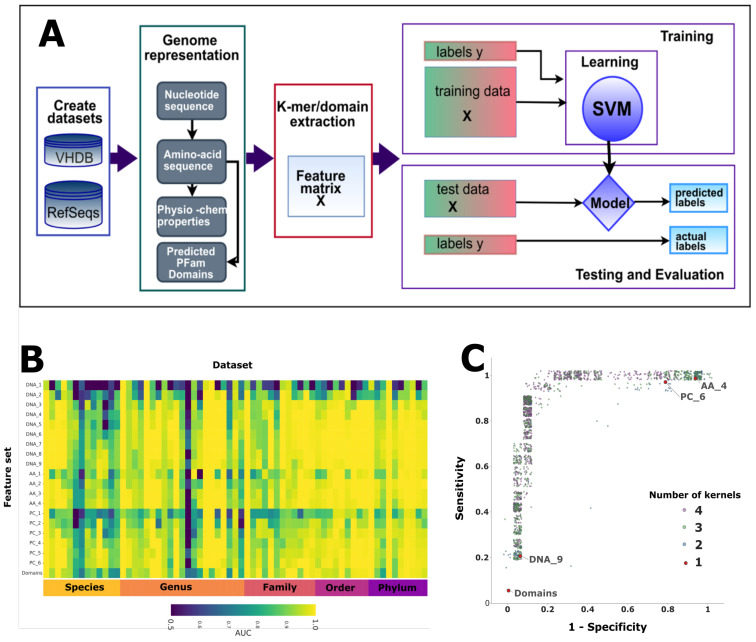
(**A**) Workflow for extracting and testing different level feature sets for predicting host taxon information. (**B**) Comparison of the results (AUC) for all the bacteria datasets for all the feature sets. (**C**) A plot of sensitivity versus specificity for kernel combination applied to one dataset. Each point represents the results for a classifier, each with a different combination of kernel weights, with the number of kernels contributing shown by the point colour.

Using both bacteria and eukaryote viruses, we compiled over a hundred binary datasets of infecting/non-infecting viruses for host taxa at all taxonomic ranks. For each dataset, we transformed the nucleotide sequences into amino acid sequences, physicochemical sequences and protein domains. Twenty feature sets were generated by extracting k-mer compositions from these sequences. We trained and tested SVM classifiers to compare the predictive capacity of each of these feature sets for each dataset (see Figure 7A). To establish whether our classifiers were learning more than a signal related directly to viral phylogeny we developed a holdout method. We removed closely related viruses from training and used these ‘holdout’ viruses to test whether our classifiers were learning host-specific signals. Finally, to demonstrate that the signals learnt from the different genome representations were complimentary, we used a kernel combination method to integrate the different levels of features and test for improvement in prediction [63].

Our results demonstrated that all four levels of genome representation were predictive of host taxonomy for both the eukaryote and bacteria taxa (see Figure 7B) and that increasing the length of the kmers improves prediction. Using our holdout method, we found that the viral genomes contain an element of non-phylogenetic signal. This indicates that machine learning is able to learn more than just phylogenetic signals and thereby has potential to improve virus host prediction. By combining features from different genome representations, we demonstrated that kernel combination not only improves prediction but can be used as a method to tune the specificity and sensitivity of a classifier (see Figure 7C).

We have shown that the four representations of the viral genomes encapsulate complimentary host-specific information. Incorporating features derived from these different representations into predictors developed for specific virus host prediction tasks should lead to improved accuracy. This will allow us to develop methods that will enable higher confidence assignments of host taxon information for the ever growing numbers of viruses with unassigned hosts.

#### 2.4.2. Recombination Networks and Endogenous Viral Anchors for High-Throughput Host Identification—Cormac M. Kinsella

**Figure 8:**
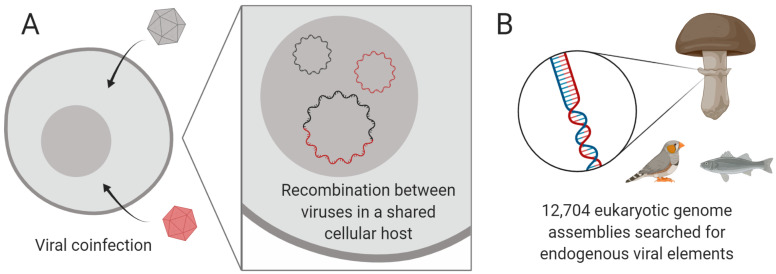
Identifying the hosts of metagenomic viruses in the Cressdnaviricota. (**A**) Recombination between single-stranded DNA viruses implies a shared cellular host, reducing the problem size from individual virus species to recombination networks. (**B**) Endogenous viral elements inside host genomes are used to anchor metagenomic viruses to specific host taxa. Created with BioRender.com.

Metagenomic sequencing has led to a surge in the number of viruses only known by their genome sequences. These sequences are a powerful resource that can greatly enhance our understanding of viral evolution and diversity; however, a vast majority of metagenomic species lack ecological data such as the cellular host [64], hampering investigation of their biological roles or medical relevance. To address this issue, computational methods for identifying hosts of metagenomic virus species are needed. To illustrate one approach, we describe the recent discovery of three circular Rep-encoding single-stranded (CRESS) DNA virus families (phylum Cressdnaviricota) in human clinical samples, and the determination of their hosts, being pathogenic human gut parasites [65]. In that study, host determination was achieved by (1) identifying recombination events between viral genomes to delimit groups overlapping in their host range, and (2) linking groups to a specific host or higher level taxon using endogenous viral elements in host genomes (see Figure 8), host small RNAs, and a case-control based examination of clinical samples.

On the basis of this work, we hypothesised that such a method could be partly extended to the wider species diversity of the Cressdnaviricota, the majority of which have no known host [66]. Members of this phylum encode at minimum a conserved replication-associated protein (Rep) and a capsid protein (Cap) with a single jelly roll fold, and these genes are frequently recombined among closely related species. Interestingly, even distantly related genomes can swap whole genetic modules to produce new recombinants [67], and we aimed to identify these long-distance events among the Cressdnaviricota. Detection of recombination events between viral genomes usually utilises nucleotide multiple sequence alignments; however, these cannot be readily generated above the family level for the Cressdnaviricota as sequence divergence levels are too large. We instead constructed a classification scheme for the Cap protein using protein sequence clustering, after which we could document the various Rep proteins co-associated with Cap groups. In parallel, we systematically searched over 12,000 eukaryotic genome assemblies for related endogenous viral elements (see Figure 8), finding over 8000 probable elements. We anticipate that these analyses will reveal numerous virus–host relationships, while unravelling aspects of CRESS DNA virus evolutionary history.

## 3. Poster Session

During a virtual poster session, twenty posters have been presented on the five topics: (1) viral evolution, (2) metagenomics and viromics, (3) viral inhibition, (4) segmented viruses and (5) tools and methods. All posters have been presented as 2-min flash presentations. Afterwards, breakout rooms have been used to group the participants topic-wise and provide the opportunity to discuss the posters in detail. Charlotte Tumescheit (University of Cambridge, Cambridge, UK) presenting a poster on CIAlign, a highly customisable command line tool to clean and interpret multiple sequence alignments, as well as German Bonilla-Rosso (Universite de Lausanne, Lausanne, Switzerland) presenting a poster on multi-strain level interactions between a diverse and persistent phage community and its hosts in the honey bee gut, received the *Best Scientific Poster* award.

## 4. EVBC Annual Meeting

The EVBC was founded in 2017 to bring together experts in virology and virus bioinformatics [1,2] and is constantly growing. Since the last annual meeting in March 2019 [4], 27 new members from 14 different countries joined the EVBC. In addition, after the conference, all speakers have been invited to join the EVBC; eight speakers followed this invitation.

During this year’s annual meeting, the EVBC Board of Directors was newly elected after the first period. We thank Martin Beer (Friedrich-Loeffler-Institut, Greifswald, Germany), Li Deng (Helmholtz Centre Munich, Germany), Philippe Le Mercier, Manja Marz, and Volker Thiel for their contribution as board members during the first three years of the EVBC and for continuing for another three years. We say goodbye to M. Palmarini, who decided to leave the board, and welcome Bas E. Dutilh (Utrecht University, Utrecht, The Netherlands), who has been newly elected.

In addition, the EVBC is happy to announce the start of the EU-funded Marie Sklodowska-Curie Innovative Training Network VIROINF (https://viroinf.eu/) which aims to understand (harmful) virus–host interactions by linking virology and bioinformatics, coordinated at the Friedrich Schiller University Jena, Germany. The consortium consists of 28 high-profile principal investigators, 17 of whom are EVBC members. The host institutions and partners are located in Austria, Belgium, France, Germany, Israel, The Netherlands, Spain, Switzerland and the United Kingdom. The VIROINF Innovative Training Network focuses on virus–host interaction by combining virus research with specifically designed bioinformatical tools to avoid infections and enable vaccinations and treatments. The objective is to train early-stage researchers in all aspects of infectious outbreaks.

## 5. Conclusions

The International Virus Bioinformatics Meeting was facing several difficulties with regards to the COVID-19 pandemic. The decision to switch to an online format was discussed thoroughly in advance by the organizers and also with the community. The main concern raised was the lack of possibilities for face-to-face discussion during virtual meetings. On the other hand, we experienced an increase in registrations after announcing the online meeting. The flexibility of listening to selected talks and not being compelled to travel (in particular, long distance) has made the meeting accessible to a broader range of scientists. Especially for people newly entering the field of virus bioinformatics, this meeting is a focal point to gain an insight into the state-of-the-art of the research landscape and to interact with researchers in the forefront as well as aspiring young scientists. The participants not only created a highly interactive scientific environment, but also a coffee break atmosphere with lively and inspiring discussions. The talks were recorded and watched afterwards by more than 100 people.

We hope that speakers summaries provided in this report will give an interesting insight into the field of virus bioinformatics and will encourage interested researchers to join us at the next International Virus Bioinformatics Meeting be held in 2022. For more information, do not hesitate to contact us via evbc@uni-jena.de.

## Figures and Tables

**Table 1:** History of the Annual Meeting of the European Virus Bioinformatics Center; renamed to International Virus Bioinformatics Meeting in 2020.

Date	Location	No. of Participants	Key Changes and Outcomes	
6–8 March 2017	Jena, Germany	~100	Founding of the center; Discussion of the role of EVBC; Election of the first Board of Directors; Insights into EU policy and funding opportunities.	
9–10 April 2018	Utrecht, The Netherlands	~120	Extending the EVBC network to include America and Asia;Discussion and design of joint projects;Insights on first applied European fund among EVBC members.	[3]
28–29 March 2019	Glasgow, United Kingdom	~110	Inclusion of contributed talks in themed sections in the scientific programme;Establishment of travel, poster and best contributed talk awards for junior scientists;Need for greater coordination and communication within the European virology community.	[4]
8–9 October 2020	virtually in Bern, Switzerland	~120	Renaming to International Virus Bioinformatics Meeting;Online format;Re-election of Board of Directors;Presentation of the newly EU-funded MSCA ITN VIROINF.

## References

[1] Ibrahim B., McMahon D.P., Hufsky F., Beer M., Deng L., Mercier P.L., Palmarini M., Thiel V., Marz M. (2018). A new era of virus bioinformatics. Virus Res..

[2] Hufsky F., Ibrahim B., Beer M., Deng L., Mercier P.L., McMahon D.P., Palmarini M., Thiel V., Marz M. (2018). Virologists-Heroes need weapons. PLoS Pathog..

[3] Ibrahim B., Arkhipova K., Andeweg A., Posada-Céspedes S., Enault F., Gruber A., Koonin E., Kupczok A., Lemey P., McHardy A. (2018). Bioinformatics Meets Virology: The European Virus Bioinformatics Center’s Second Annual Meeting. Viruses.

[4] Hufsky F., Ibrahim B., Modha S., Clokie M.R.J., Deinhardt-Emmer S., Dutilh B.E., Lycett S., Simmonds P., Thiel V., Abroi A. (2019). The Third Annual Meeting of the European Virus Bioinformatics Center. Viruses.

[5] Watts J.M., Dang K.K., Gorelick R.J., Leonard C.W., Bess J.W., Swanstrom R., Burch C.L., Weeks K.M. (2009). Architecture and secondary structure of an entire HIV-1 RNA genome. Nature.

[6] Pedersen J.S., Forsberg R., Meyer I.M., Hein J. (2004). An evolutionary model for protein-coding regions with conserved RNA structure. Mol. Biol. Evol..

[7] Pedersen J.S., Meyer I.M., Forsberg R., Simmonds P., Hein J. (2004). A comparative method for finding and folding RNA secondary structures within protein-coding regions. Nucleic Acids Res..

[8] Bogdanow B., Wang X., Eichelbaum K., Sadewasser A., Husic I., Paki K., Budt M., Hergeselle M., Vetter B., Hou J. (2019). The dynamic proteome of influenza A virus infection identifies M segment splicing as a host range determinant. Nat. Commun..

[9] Lai D., Proctor J.R., Meyer I.M. (2013). On the importance of cotranscriptional RNA structure formation. RNA.

[10] Proctor J.R., Meyer I.M. (2013). COFOLD: An RNA secondary structure prediction method that takes co-transcriptional folding into account. Nucleic Acids Res..

[11] Meyer I.M. (2017). In silico methods for co-transcriptional RNA secondary structure prediction and for investigating alternative RNA structure expression. Methods.

[12] Wiebe N.J.P., Meyer I.M. (2010). TRANSAT—Method for detecting the conserved helices of functional RNA structures, including transient, pseudo-knotted and alternative structures. PLoS Comput. Biol..

[13] Zhu J.Y.A., Steif A., Proctor J.R., Meyer I.M. (2013). Transient RNA structure features are evolutionarily conserved and can be computationally predicted. Nucleic Acids Res..

[14] Zhu J.Y.A., Meyer I.M. (2015). Four RNA families with functional transient structures. RNA Biol..

[15] Lai D., Proctor J.R., Zhu J.Y.A., Meyer I.M. (2012). R-CHIE: A web server and R package for visualizing RNA secondary structures. Nucleic Acids Res..

[16] Lu Z., Zhang Q.C., Lee B., Flynn R.A., Smith M.A., Robinson J.T., Davidovich C., Gooding A.R., Goodrich K.J., Mattick J.S. (2016). RNA Duplex Map in Living Cells Reveals Higher-Order Transcriptome Structure. Cell.

[17] Aw J.G.A., Shen Y., Wilm A., Sun M., Lim X.N., Boon K.L., Tapsin S., Chan Y.S., Tan C.P., Sim A.Y.L. (2016). In vivo Mapping of Eukaryotic RNA Interactomes Reveals Principles of Higher-Order Organization and Regulation. Mol. Cell.

[18] Sharma E., Sterne-Weiler T., O’Hanlon D., Blencowe B.J. (2016). Global Mapping of Human RNA-RNA Interactions. Mol. Cell.

[19] Stefanov S.R., Meyer I.M. (2018). Deciphering the Universe of RNA Structures and trans RNA–RNA Interactions of Transcriptomes in vivo: From Experimental Protocols to Computational Analyses. RNA Technologies.

[20] Lai D., Meyer I.M. (2014). e-RNA: A collection of web servers for comparative RNA structure prediction and visualisation. Nucleic Acids Res..

[21] Tsybulskyi V., Mounir M., Meyer I.M. (2020). R-chie: A web server and R package for visualizing cis and trans RNA–RNA, RNA–DNA and DNA–DNA interactions. Nucleic Acids Res..

[22] Wolf Y.I., Kazlauskas D., Iranzo J., Lucía-Sanz A., Kuhn J.H., Krupovic M., Dolja V.V., Koonin E.V. (2018). Origins and Evolution of the Global RNA Virome. mBio.

[23] Olendraite I., Lukhovitskaya N.I., Porter S.D., Valles S.M., Firth A.E. (2017). Polycipiviridae: A proposed new family of polycistronic picorna-like RNA viruses. J. Gen. Virol..

[24] Ingolia N.T., Brar G.A., Rouskin S., McGeachy A.M., Weissman J.S. (2012). The ribosome profiling strategy for monitoring translation in vivo by deep sequencing of ribosome-protected mRNA fragments. Nat. Protoc..

[25] Ingolia N.T., Ghaemmaghami S., Newman J.R.S., Weissman J.S. (2009). Genome-wide analysis *in vivo* of translation with nucleotide resolution using ribosome profiling. Science.

[26] Fang Y., Treffers E.E., Li Y., Tas A., Sun Z., van der Meer Y., de Ru A.H., van Veelen P.A., Atkins J.F., Snijder E.J. (2012). Efficient -2 frameshifting by mammalian ribosomes to synthesize an additional arterivirus protein. Proc. Natl. Acad. Sci. USA.

[27] Li Y., Treffers E.E., Napthine S., Tas A., Zhu L., Sun Z., Bell S., Mark B.L., van Veelen P.A., van Hemert M.J. (2014). Transactivation of programmed ribosomal frameshifting by a viral protein. Proc. Natl. Acad. Sci. USA.

[28] Napthine S., Treffers E.E., Bell S., Goodfellow I., Fang Y., Firth A.E., Snijder E.J., Brierley I. (2016). A novel role for poly(C) binding proteins in programmed ribosomal frameshifting. Nucleic Acids Res..

[29] Finch L.K., Ling R., Napthine S., Olspert A., Michiels T., Lardinois C., Bell S., Loughran G., Brierley I., Firth A.E. (2015). Characterization of Ribosomal Frameshifting in Theiler’s Murine Encephalomyelitis Virus. J. Virol..

[30] Hill C.H., Cook G., Napthine S., Kibe A., Brown K., Caliskan N., Firth A.E., Graham S.C., Brierley I. (2020). Structural and molecular basis for protein-stimulated ribosomal frameshifting in Theiler’s murine encephalomyelitis virus. bioRxiv.

[31] Breitbart M., Bonnain C., Malki K., Sawaya N.A. (2018). Phage puppet masters of the marine microbial realm. Nat. Microbiol..

[32] Brum J.R., Sullivan M.B. (2015). Rising to the challenge: Accelerated pace of discovery transforms marine virology. Nat. Rev. Microbiol..

[33] Ogilvie L.A., Jones B.V. (2015). The human gut virome: A multifaceted majority. Front. Microbiol..

[34] Rodriguez-Brito B., Li L., Wegley L., Furlan M., Angly F., Breitbart M., Buchanan J., Desnues C., Dinsdale E., Edwards R. (2010). Viral and microbial community dynamics in four aquatic environments. ISME J..

[35] Suttle C.A. (2005). Viruses in the sea. Nature.

[36] Zimmerman A.E., Howard-Varona C., Needham D.M., John S.G., Worden A.Z., Sullivan M.B., Waldbauer J.R., Coleman M.L. (2020). Metabolic and biogeochemical consequences of viral infection in aquatic ecosystems. Nat. Rev. Microbiol..

[37] Feiner R., Argov T., Rabinovich L., Sigal N., Borovok I., Herskovits A.A. (2015). A new perspective on lysogeny: Prophages as active regulatory switches of bacteria. Nat. Rev. Microbiol..

[38] Paez-Espino D., Roux S., Chen I.M.A., Palaniappan K., Ratner A., Chu K., Huntemann M., Reddy T.B.K., Pons J.C., Llabrés M. (2019). IMG/VR v.2.0: An integrated data management and analysis system for cultivated and environmental viral genomes. Nucleic Acids Res..

[39] Roux S., Brum J.R., Dutilh B.E., Sunagawa S., Duhaime M.B., Loy A., Poulos B.T., Solonenko N., Lara E., Poulain J. (2016). Ecogenomics and potential biogeochemical impacts of globally abundant ocean viruses. Nature.

[40] Emerson J.B., Roux S., Brum J.R., Bolduc B., Woodcroft B.J., Jang H.B., Singleton C.M., Solden L.M., Naas A.E., Boyd J.A. (2018). Host-linked soil viral ecology along a permafrost thaw gradient. Nat. Microbiol..

[41] Henry K.A., Arbabi-Ghahroudi M., Scott J.K. (2015). Beyond phage display: Non-traditional applications of the filamentous bacteriophage as a vaccine carrier, therapeutic biologic, and bioconjugation scaffold. Front. Microbiol..

[42] Salmond G.P.C., Fineran P.C. (2015). A century of the phage: Past, present and future. Nat. Rev. Microbiol..

[43] Roux S., Adriaenssens E.M., Dutilh B.E., Koonin E.V., Kropinski A.M., Krupovic M., Kuhn J.H., Lavigne R., Brister J.R., Varsani A. (2019). Minimum Information about an Uncultivated Virus Genome (MIUViG). Nat. Biotechnol..

[44] Roux S., Krupovic M., Daly R.A., Borges A.L., Nayfach S., Schulz F., Sharrar A., Matheus Carnevali P.B., Cheng J.F., Ivanova N.N. (2019). Cryptic inoviruses revealed as pervasive in bacteria and archaea across Earth’s biomes. Nat. Microbiol..

[45] Roux S. (2019). A Viral Ecogenomics Framework to Uncover the Secrets of Nature’s “Microbe Whisperers”. mSystems.

[46] Roux S., Brum J.R. (2019). A viral reckoning: Viruses emerge as essential manipulators of global ecosystems. Environ. Microbiol. Rep..

[47] Emerson J.B. (2019). Soil Viruses: A New Hope. mSystems.

[48] Kortright K.E., Chan B.K., Koff J.L., Turner P.E. (2019). Phage Therapy: A Renewed Approach to Combat Antibiotic-Resistant Bacteria. Cell Host Microbe.

[49] Huson D.H., Beier S., Flade I., Górska A., El-Hadidi M., Mitra S., Ruscheweyh H.J., Tappu R. (2016). MEGAN Community Edition—Interactive Exploration and Analysis of Large-Scale Microbiome Sequencing Data. PLoS Comput. Biol..

[50] Ondov B.D., Bergman N.H., Phillippy A.M. (2011). Interactive metagenomic visualization in a Web browser. BMC Bioinf..

[51] Eren A.M., Esen Ö.C., Quince C., Vineis J.H., Morrison H.G., Sogin M.L., Delmont T.O. (2015). Anvi’o: An advanced analysis and visualization platform for ’omics data. PeerJ.

[52] Posada-Céspedes S., Seifert D., Topolsky I., Metzner K.J., Beerenwinkel N. (2020). V-pipe: A computational pipeline for assessing viral genetic diversity from high-throughput sequencing data. bioRxiv.

[53] Kuipers J., Batavia A.A., Jablonski K.P., Bayer F., Borgsmüller N., Dondi A., Drăgan M.A., Ferreira P., Jahn K., Lamberti L. (2020). Within-patient genetic diversity of SARS-CoV-2. bioRxiv.

[54] Käfer S., Paraskevopoulou S., Zirkel F., Wieseke N., Donath A., Petersen M., Jones T.C., Liu S., Zhou X., Middendorf M. (2019). Re-assessing the diversity of negative strand RNA viruses in insects. PLoS Pathog..

[55] Misof B., Liu S., Meusemann K., Peters R.S., Donath A., Mayer C., Frandsen P.B., Ware J., Flouri T., Beutel R.G. (2014). Phylogenomics resolves the timing and pattern of insect evolution. Science.

[56] Eddy S.R. (2011). Accelerated Profile HMM Searches. PLoS Comput. Biol..

[57] Tommaso P.D., Chatzou M., Floden E.W., Barja P.P., Palumbo E., Notredame C. (2017). Nextflow enables reproducible computational workflows. Nat. Biotechnol..

[58] McInnes L., Healy J., Melville J. (2018). Umap: Uniform manifold approximation and projection for dimension reduction. arXiv.

[59] McInnes L., Healy J., Astels S. (2017). HDBSCAN: Hierarchical density based clustering. J. Open Source Softw..

[60] Katoh K., Standley D.M. (2013). MAFFT Multiple Sequence Alignment Software Version 7: Improvements in Performance and Usability. Mol. Biol. Evol..

[61] Price M.N., Dehal P.S., Arkin A.P. (2010). FastTree 2—Approximately Maximum-Likelihood Trees for Large Alignments. PLoS ONE.

[62] Fu L., Niu B., Zhu Z., Wu S., Li W. (2012). CD-HIT: Accelerated for clustering the next-generation sequencing data. Bioinformatics.

[63] Young F., Rogers S., Robertson D.L. (2020). Predicting host taxonomic information from viral genomes: A comparison of feature representations. PLoS Comput. Biol..

[64] Simmonds P., Adams M.J., Benkő M., Breitbart M., Brister J.R., Carstens E.B., Davison A.J., Delwart E., Gorbalenya A.E., Harrach B. (2017). Consensus statement: Virus taxonomy in the age of metagenomics. Nat. Rev. Microbiol..

[65] Kinsella C.M., Bart A., Deijs M., Broekhuizen P., Kaczorowska J., Jebbink M.F., van Gool T., Cotten M., van der Hoek L. (2020). *Entamoeba* and *Giardia* parasites implicated as hosts of CRESS viruses. Nat. Commun..

[66] Kazlauskas D., Varsani A., Krupovic M. (2018). Pervasive Chimerism in the Replication-Associated Proteins of Uncultured Single-Stranded DNA Viruses. Viruses.

[67] Roux S., Enault F., Bronner G., Vaulot D., Forterre P., Krupovic M. (2013). Chimeric viruses blur the borders between the major groups of eukaryotic single-stranded DNA viruses. Nat. Commun..

